# Scavengers of Oxygen-Derived Free Radicals: A New Approach to the Problem of Pancreatitis-Induced Abdominal Pain

**DOI:** 10.1155/1991/15639

**Published:** 1991

**Authors:** Aws S. Salim

**Affiliations:** The Department of Surgery Ward 6 Stobhill General Hospital Glasgow G21 3UW UK


*HPB Surgery*, 1991, Vol. 4, pp 331–333
Reprints available directly from the publisher
Photocopying permitted by license only
© 1991 Harwood Academic Publishers GmbH
Printed in the United Kingdom

### LETTER TO THE EDITOR

### SCAVENGERS OF OXYGEN-DERIVED FREE RADICALS

### A New Approach to the Problem of Pancreatitis-induced Abdominal Pain

The abdominal pain produced by chronic pancreatitis may be very difficult to treat. Oxygen-derived free radicals, such as the superoxide anion and hydroxyl radical, are cytotoxic and promote tissue damage[1,2]. These radicals injure cellular membranes and release the intracellular components, e.g. lysosomal enzymes, that can lead to further tissue damage[3,4]. In addition, the radicals cause degradation of hyaluronic acid, the principal component of epithelial basement membranes and crosslink lipids, proteins and nucleic acids, thus, promoting tissue injury[3,4]. Recently, oxygen-derived free radicals have been shown to play an important role in the pathogenesis of acute pancreatitis in experimental animals and scavenging them ameliorates the severity of this pancreatitis[5,6]. The clinical significance of such observations is not known and this communication is the first report on the influence of radical scavengers on the abdominal pain produced by chronic alcoholic pancreatitis.

Six consecutive males (age range 38–57, mean 42) who were known to have alcohol-induced chronic pancreatitis were admitted to hospital (The Department of Surgery, Perth Royal Infirmary, U.K.) with a less than 2 hours' history of sharp, severe and constant epigastric pain radiating to the back and associated with nausea and/or vomiting. Chronic pancreatitis was diagnosed (speckled calcification visible on plain X-ray and CAT-scan, disorganised duct pattern on pancreatography, fibrosis and chronic inflammation seen histologically) 2 to 3 years before this admission and since then the clinical course has been punctuated by a series of acute illnesses identical to that described above and resulting in hospitalization at least twice a year during which time pain lasts for a minimum of 3 days despite active treatment. These subjects had impaired pancreatic exocrine secretion manifested by various degrees of steatorrhoea, for which they were given pancreatic enzyme supplements (with an $H_2$-receptor antagonist), and weight loss, despite a good apetite. Three patients had diabetes and none had any evidence of biliary disease.

On examination, all patients were distressed, had epigastric tenderness with various degrees of guarding, leucocytosis ($9.33 \pm 0.8 \times 10^9$/L, mean ± SEM), and a serum amylase of 398 ± 11.7 1.U/L (mean ± SEM; normal value 70 – 300 1.U/L). An informed consent was obtained from all cases then they were completely fasted, intravenously hydrated, and given intramuscular pethidine (initially 100

mg to be followed by 50 mg every 4 hours until complete pain relief), hyoscine butylbromide (10 mg every 8 hours) and metoclopramide (10 mg every 8 hours). The diabetic patients were treated with soluble insulin. Endoscopy was carried out within 6 hours after admission to exclude other upper GIT disorders, plain abdominal X-rays were taken to exclude visceral perforation, and an abdominal ultrasound was performed to rule out biliary calculi.

Twelve hours after admission, the epigastric pain remained unaltered despite 250 mg of pethidine, and 3 patients were instituted on 5 ml of 2% solution (100 mg) of allopurinol (prepared by dissolving the powder in double distilled water containing the molar equivalent of 0.1 M NaOH) per rectum every 8 hours while the remaining 3 patients were similarly treated with 5 ml of 10% (500 mg) dimethyl sulphoxide (DMSO, pharmaceutical grade). During the first 12 hours after commencing these agents, all patients became completely pain free. Twenty-four hours after hospitalization, the pethidine, hyoscine and metoclopramide were stopped and patients were gradually re-introduced to oral fluids. The epigastric tenderness was still present in all patients at this stage. Over the following 12 hours all the patients remained asymptomatic despite persistent epigastric tenderness. On the third day after admission all patients were comfortably tolerating free fluids by mouth. Consequently, the intravenous line was disconnected, the allopurinol and DMSO were stopped and the intake of solid food with pancreatic enzyme supplements (and $H_2$-receptor antagonists) recommenced. The epigastric tenderness disappeared by the fourth day after admission and all cases continued to be asymptomatic, therefore they were discharged home.

DMSO scavenges hydroxyl radicals and allopurinol is also a potent scavenger of these radicals besides inhibiting the enzyme xanthine oxidase, which is responsible for the formation of superoxide radicals[4,7]. The role of these radicals in the pathogenesis of acute pancreatitis in experimental animals has been confirmed. Relative to the previous clinical experiences and pain history of the patients studied, the results presented suggest that scavengers of oxygen-derived free radicals accelerate the control of the pancreatitis-induced abdominal pain. Since the recurrent episodes of chronic pancreatitis are associated with an inflammatory process within the gland and since oxyradicals mediate inflammation[8,9,10], it appears that these radicals are directly implicated in the mechanism of the acute episodes of alcohol induced chronic pancreatitis. The results also suggest that scavenging oxyradicals ameliorates the severity of the abdominal pain associated with these episodes. The similarity in efficacy between allopurinol and DMSO and the fact that the only action they share is scavenging oxyradicals supports the suggestions raised by this study.

*(Accepted by S. Bengmark 7 May 1991)*

### *References*

1. Butterfield, J.D. and McGraw, C.P. (1978) Free radical pathology. *Stroke*, **9**, 443–445
2. Salim, A.S. (1989) Role of oxygen-derived free radicals in the mechanism of chronic gastric ulceration in the rat: Implications for cytoprotection. *Digestion*, **43**, 113–119
3. Del Maestro, R.F., Thaw, H.H., Bjork, J., Planker, M. and Arfors, K.E. (1980) Free radicals as mediators of tissue injury. *Acta Physiol. Scand.*, (Suppl.) **492**, 43–57
4. Itoh, M. and Guth, P.H. (1985) Role of oxygen-derived free radicals in hemorrhagic shock-induced gastric lesions in the rat. *Gastroenterology*, **88**, 1162–1167

5. Sanfey, H., Bulkley, G.B. and Cameron, J.L. (1985) The pathogenesis of acute pancreatitis. The source and role of oxygen-derived free radicals in three different experimental models. *Ann. Surg.*, **201**, 633–639
6. Nonaka, A., Manabe, T., Tamura, K., Asano, N., Imanishi, K. and Tobe, T. (1989) Changes in xanthine oxidase, lipid peroxide and superoxide dismutase in mouse acute pancreatitis. *Digestion*, **43**, 41–46
7. Moorhouse, P.C., Grootveld, M., Halliwell, B., Quinlan, J.G. and Gutteridge, J.M.C. (1987) Allopurinol and oxypurinol are hydroxyl radical scavengers. *FEBS Lett.*, **213**, 23–28
8. Salim, A.S. (1989) Scavenging free radicals to prevent stress-induced gastric mucosal injury. *Lancet*, **II**, 1390
9. Salim, A.S. (1990) Role of oxygen-derived free radicals in the mechanism of acute and chronic duodenal ulceration in the rat. *Dig. Dis. Sci.*, **35**, 73–79
10. Salim, A.S. (1990) The significance of removing oxygen-derived free radicals in the treatment of acute and chronic duodenal ulceration in the rat. *J. Pharm. Pharmacol.*, **42**, 64–67

Aws S. Salim
The Department of Surgery,
Ward 6,
Stobhill General Hospital
Glasgow G21 3UW, UK

